# A Mediterranean-type diet is associated with better metabolic profile in urban Polish adults: Results from the HAPIEE study

**DOI:** 10.1016/j.metabol.2015.02.007

**Published:** 2015-06

**Authors:** Giuseppe Grosso, Urszula Stepaniak, Agnieszka Micek, Roman Topor-Mądry, Denes Stefler, Krystyna Szafraniec, Martin Bobak, Andrzej Pająk

**Affiliations:** a Department of Clinical and Molecular Biomedicine, Section of Pharmacology and Biochemistry, University of Catania, Catania, Italy; b Department of Epidemiology and Population Studies, Jagiellonian University Medical College, Krakow, Poland; c Department of Epidemiology and Public Health, University College of London, London, United Kingdom

**Keywords:** BMI, body mass index, CVD, cardiovascular disease, DBP, diastolic blood pressure, FA, fatty acids, FFQ, Food Frequency Questionnaire, FPG, fasting plasma glucose, HAPIEE, Health, Alcohol and Psychosocial factors In Eastern Europe, HDL-c, high-density lipoprotein cholesterol, LDL-c, low-density lipoprotein cholesterol, MedTypeDiet, Mediterranean-type diet, MetS, metabolic syndrome, MUFA, monounsaturated fatty acids, PUFA, polyunsaturated fatty acids, SBP, systolic blood pressure, TC, total cholesterol, TG, triglycerides, WC, waist circumference, Mediterranean diet, Metabolic syndrome, Obesity, Hypertension, Diabetes

## Abstract

**Objective:**

The aim of this study was to evaluate the relationship between adherence to a Mediterranean-type diet and metabolic syndrome (MetS) in the Polish arm of the Health, Alcohol and Psychosocial factors In Eastern Europe (HAPIEE) cohort study.

**Materials/methods:**

A cross-sectional survey including 8821 adults was conducted in Krakow, Poland. Food intake was evaluated through a validated food frequency questionnaire and adherence to the dietary pattern was assessed using a score specifically developed for non-Mediterranean countries (MedTypeDiet score). Linear and logistic regression models were performed to estimate beta and odds ratios (ORs) and 95% confidence intervals (CIs), respectively.

**Results:**

Significant associations between the MedTypeDiet score and waist circumference (β = − 0.307 ± 0.239 cm), systolic blood pressure (β = − 0.440 ± 0.428 mmHg), and triglycerides (β = − 0.021 ± 0.016 mmol/L) were observed. After multivariable adjustment, individuals in the highest quartile of the score were less likely to have MetS, central obesity, high triglycerides, and hypertension. Increase of one standard deviation of the score was associated with 7% less odds of having MetS (OR 0.93, 95% CI: 0.88, 0.97). When analyzing the relation of single components of the MedTypeDiet score, wine, dairy products, and the total unsaturated:saturated fatty acids ratio were associated with MetS.

**Conclusions:**

Adherence to a Mediterranean-like diet may decrease the risk of MetS also among non-Mediterranean populations.

## 1 Introduction

The metabolic syndrome (MetS) is characterized by a cluster of metabolic and physiological perturbations that include abdominal obesity, dyslipidemia, elevated blood pressure, and elevated fasting glucose [1]. The occurrence of these clustered conditions has been widely demonstrated to increase the risk of cardiovascular disease (CVD) [2]. The prevalence of MetS in European adults has been estimated at 20–30% overall, with higher prevalence in southern and eastern countries [3]. There is evidence that MetS has become more widespread over the last decade, probably influenced by increases in obesity prevalence [3]. Weight status, together with physical activity and diet, has been reported to be among the main environmental determinants of the MetS [4]. However, there is no universal approach to stop, at least in part, the rise of this condition burden.

Adherence to healthy dietary patterns, for instance rich in fruit and vegetable and scarce in red meat, has been shown to prevent components of the MetS. Among the most studied dietary pattern, the Mediterranean diet demonstrated to be associated with several health outcomes [5,6]. Many of the studies exploring the protective effects of the Mediterranean diet against the MetS reported mostly univocal results [7], but it is noteworthy that most of them were conducted on Mediterranean populations [8–13]. Moreover, findings from these studies demonstrated an increasing higher proportion of southern populations not adherent to this dietary pattern due to the nutrition transition phenomenon, in most of cases associated with unhealthy outcomes and reasonably cause, at least in part, of risen burden of MetS in such areas [14]. Although there is no consensus in the definition of the Mediterranean diet due to country-specific features even among Mediterranean countries themselves, the most common characteristics are represented by consumption of a large quantity of plant-derived foods (fruit, vegetables, and legumes), cereals (especially whole-grain), and fish, low intake of meat and dairy products, daily intake of olive oil and nuts as main source of fat, and moderate intake of wine (especially red wine) during meals [15]. Its benefits are supposed to depend on its high content of antioxidants and fibers, with fish, nuts and olive oil that ensure a high intake of polyunsaturated fatty acids (PUFA) and monounsaturated fatty acids (MUFA), associated with a low intake of trans-fatty acids from meat and sweets [15]. Since there is evidence that this dietary pattern protects against CVD and cardiovascular risk factors, it is of particular interest to assess if epidemiological evidence retrieved in Mediterranean populations can be demonstrated also in non-Mediterranean countries. Previous studies attempting the application of tools evaluating the adherence to a Mediterranean-type diet to non-Mediterranean countries were mostly focused on weight change [16–18] and studies associating the Mediterranean diet to overall clustered risk factors included in the MetS are limited to two studies performed on US population [19,20]. To the best of our knowledge, the association between Mediterranean diet and MetS and its components has never been explored in European non-Mediterranean countries.

The aim of the present study was to evaluate the association between the level of adherence to a Mediterranean-type dietary pattern and the metabolic profile in the Polish arm of the Health, Alcohol and Psychosocial factors In Eastern Europe (HAPIEE) study. We developed a specific adherence index taking into account both the dietary habits of non-Mediterranean populations and specific components that confer healthy characteristics to this dietary pattern, and related it with MetS and its components.

## 2 Methods

### 2.1 Study population

The HAPIEE cohort study is a multicenter prospective cohort study investigating the associations of biological, dietary, lifestyle, and environmental factors with CVD and aging processes [21]. The present investigation included individuals of the Polish arm of the HAPIEE study. The criteria for sample selection and the methods are reported in detail elsewhere [21]. Briefly, a random sample of 10,728 adults (aged 45–69 years) was recruited at the baseline survey conducted in 2002–2005 (participation rate of 59%) in the urban area of Krakow, Poland. The participants completed a structured questionnaire and were invited at clinical examination. We included in the analysis those who attended the physical examination and provided blood samples (n = 9050), whereas we excluded participants who left more than 50% food items blank in food-frequency questionnaires (FFQs) or reported implausible energy intake (< 500/> 4000 kcal/d for females and < 800/> 5000 kcal/d for males, n = 14) and those who had missing values for any of the covariates (n = 215). The final sample comprised of 8821 participants (51.4% female). The study protocol was approved by the ethics committee at University College London, UK, and by the bioethics committee of the Jagiellonian University (no. KE/99/03/B/284 2). Participants provided informed written consent before joining the study.

### 2.2 Demographic, lifestyle and clinical measurements

Socio-demographic and lifestyle characteristics included age, gender, educational and occupational level, smoking and alcohol drinking habits. Educational level was categorized as (i) low (primary/secondary), (ii) medium (high school), and (ii) high (university). Occupational level was categorized as (i) low (unskilled/unemployed workers), (ii) medium (partially skilled workers), and (iii) high (skilled workers). Physical activity level was calculated by taking into account energy expenditure from activities both at work and leisure time, frequency (times per week converted in daily), duration (minutes per time), and intensity (expended calories). Intensity was categorized in light [expended energy < 16.7 kJ (< 4 kcal)/min], moderate [expended energy 16.7–29.3 kJ (4–7 kcal)/min], and high [expended energy > 29.3 kJ (7 kcal)/min]. A combined score by multiplying weekly frequency, duration, and intensity of physical activity was calculated and individuals gradated in qualitative terms such as (i) low, (ii) moderately, and (iii) highly active. Individuals were categorized according to their smoking status as non-smoker or current smoker. Alcohol consumption was categorized as (i) up to or (ii) more than 12 g/day.

The physical examination included measurement of height, weight, waist circumference (WC) and blood pressure using standard procedures [21]. Body mass index (BMI) was calculated according the formula weight (kg)/height^2^ (m). WC was measured midway between the 12th rib and the iliac crest. Blood pressure was measured three times at the end of the physical examination and the final value was the mean among the three measurements.

### 2.3 Definition of MetS

MetS was defined according to the International Diabetes Federation definition [22], as having central obesity (WC ≥ 90 cm in men and ≥ 80 cm in women) and any two of the following: (i) triglycerides (TG) > 150 mg/dL (1.7 mmol/L), or specific treatment for this lipid abnormality; (ii) high-density lipoprotein cholesterol (HDL-c) < 40 mg/dL (1.03 mmol/L) in males, < 50 mg/dL (1.29 mmol/L) in females, or specific treatment for this lipid abnormality; (iii) systolic blood pressure (SBP) > 130 or diastolic blood pressure (DBP) > 85 mm Hg, or treatment of previously diagnosed hypertension; (iv) fasting plasma glucose (FPG) > 100 mg/dL (5.6 mmol/L), or previously diagnosed type 2 diabetes or treatment of previously diagnosed diabetes.

### 2.4 Dietary assessment

Dietary data were collected by using a food frequency questionnaire (FFQ) based on the tool developed by Willett et al. [23]. The FFQs consisted of a 148 food- and drink-item. An instruction manual which included photographs to facilitate the estimation of portion sizes was used. Participants were asked how often, on average, they had consumed that amount of the item during the last three months, with nine responses ranging from “never or less than once per month” to “six or more times per day”. Moreover, participants were asked to include additional foods and frequency of consumption by manual entry if the food was not included in the items of the FFQ. For each participant, daily intakes (in grams) of vegetables, legumes, fruits and nuts, cereals, fish and seafood, dairy products, meat and meat products (including red meat, poultry, and processed meat), and alcohol were estimated.

### 2.5 Mediterranean-type diet score

A score indicating the degree of adherence to the Mediterranean diet was built taking into account previous instruments [24,25]. However, we provided some slight modifications in order to better apply this tool to non-Mediterranean countries. Specifically, we used the approach suggested by Panagiotakos et al. [24], according which an individual rating (from 0 to 5) to weekly consumption of the food groups mainly inherent with the characteristics of the Mediterranean dietary pattern was given. Foods included in our tool were: (i) non-refined cereals (wholegrain bread and pasta, brown rice, etc.), (ii) potatoes, (iii) Fruits, (iv) vegetables, (v) legumes, (vi) fish, and (vii) nuts and seeds consumption (this last not included in the original tool). Contribution of olive oil in non-Mediterranean countries is highly debatable due to its scarce consumption. Its health benefits are supposed to be associated, at least in part, with its content in MUFA, but it has been reported that in non-Mediterranean countries, MUFA may reflect, to a large extent, the consumption of animal fat. Therefore, the ratio of the sum of MUFA and PUFA to saturated fatty acids (FA) was calculated and a score ranging from 0 to 5 was assigned according to sex-specific quantiles, as previously suggested [16]. A reverse scale (from 5 to 0) was assigned for consumption of those foods presumed to be not typical of the Mediterranean pattern: (viii) meat and meat products, (ix) poultry, and (x) full-fat dairy products (such as cheese, yogurt, milk). In our variant of the tool, evaluation of usual wine intake was preferred to total alcohol intake, as in non-Mediterranean countries contribution to total alcohol intake would rely mainly on other alcoholic drinks (i.e., liqueurs, and spirits). Thus, wine intake was evaluated with a non-monotonic scoring system (i.e., assigning a score of 5 for consumption of < 300 ml/d, a score of 0 for no consumption or for consumption of > 700 ml/d, and a score of 4 to 1 for consumption of 600–700, 500–600, 400–500 and 300–400 ml/d, respectively). According to previous studies evaluating the Mediterranean diet in non-Mediterranean countries, it has been suggested that a particular attention should be paid on quantities of each of the diet components. We considered as daily cut-off point of absolute values of single component to be reached in order to describe adherence to the Mediterranean diet (≥ 4 points per food group) the change-points proposed in the relationship between food groups in the Mediterranean diet and overall mortality [25] and CVD [26]. The total score could range from 0 to 60 and higher values indicate greater adherence to the Mediterranean diet. The MedTypeDiet Score components and their scoring criteria are shown in the Supplementary Appendix (Table A1).

A score indicating the degree of adherence to the Mediterranean diet was built taking into account previous instruments [24,25]. However, we provided some slight modifications in order to better apply this tool to non-Mediterranean countries. Specifically, we used the approach suggested by Panagiotakos et al. [24], according which an individual rating (from 0 to 5) to weekly consumption of the food groups mainly inherent with the characteristics of the Mediterranean dietary pattern was given. Foods included in our tool were: (i) non-refined cereals (wholegrain bread and pasta, brown rice, etc.), (ii) potatoes, (iii) Fruits, (iv) vegetables, (v) legumes, (vi) fish, and (vii) nuts and seeds consumption (this last not included in the original tool). Contribution of olive oil in non-Mediterranean countries is highly debatable due to its scarce consumption. Its health benefits are supposed to be associated, at least in part, with its content in MUFA, but it has been reported that in non-Mediterranean countries, MUFA may reflect, to a large extent, the consumption of animal fat. Therefore, the ratio of the sum of MUFA and PUFA to saturated fatty acids (FA) was calculated and a score ranging from 0 to 5 was assigned according to sex-specific quantiles, as previously suggested [16]. A reverse scale (from 5 to 0) was assigned for consumption of those foods presumed to be not typical of the Mediterranean pattern: (viii) meat and meat products, (ix) poultry, and (x) full-fat dairy products (such as cheese, yogurt, milk). In our variant of the tool, evaluation of usual wine intake was preferred to total alcohol intake, as in non-Mediterranean countries contribution to total alcohol intake would rely mainly on other alcoholic drinks (i.e., liqueurs, and spirits). Thus, wine intake was evaluated with a non-monotonic scoring system (i.e., assigning a score of 5 for consumption of < 300 ml/d, a score of 0 for no consumption or for consumption of > 700 ml/d, and a score of 4 to 1 for consumption of 600–700, 500–600, 400–500 and 300–400 ml/d, respectively). According to previous studies evaluating the Mediterranean diet in non-Mediterranean countries, it has been suggested that a particular attention should be paid on quantities of each of the diet components. We considered as daily cut-off point of absolute values of single component to be reached in order to describe adherence to the Mediterranean diet (≥ 4 points per food group) the change-points proposed in the relationship between food groups in the Mediterranean diet and overall mortality [25] and CVD [26]. The total score could range from 0 to 60 and higher values indicate greater adherence to the Mediterranean diet. The MedTypeDiet Score components and their scoring criteria are shown in the Supplementary Appendix (Table A1).

### 2.6 Statistical analysis

Analyses were performed using SPSS software v. 17.0 (Chicago, IL). Continuous variables are presented as means and standard deviations (SDs), categorical variables as frequencies and percentages. Variables were examined for normality distribution (Kolmogorov–Smirnov). Chi-square test was used for comparisons of categorical variables, ANOVA or Kruskal–Wallis test was used for continuous variables according to distribution. We considered the score as exposure variable, both as quartiles (Q1–Q4) and variation by 1-SD. Multivariable linear regression models were performed to assess the relationship between metabolic parameters (BMI, WC, HDL-c, FPG, SBP, DBP, low-density lipoprotein cholesterol [LDL-c], total cholesterol [TC] and the TC:HDL ratio) as dependent variables and 1-SD increase of the Mediterranean-type diet score as continuous variable, adjusting for age, gender, education, occupation, physical activity, smoking status, and total energy intake. Results from the regression models were presented as β-coefficients and standard error. Normality of the standardized residuals was assessed using the Shapiro–Wilk test. The assumption of linearity for the continuous independent variables and of the variance of the standardized residuals being constant was assessed through plotting the residuals against the fitted values. Finally, odds ratios (ORs) and 95% confidence intervals (CIs) assessing the association of both categorized score groups and 1-SD increase of the score, with having MetS or its individual components, were calculated by multivariable logistic regression models. Gender-specific analyses were also conducted to take into account the natural differences in body composition and caloric needs between men and women. To test the role of individual score components, further logistic regression models were performed in order to evaluate the potential association with MetS of each component of the score. Finally, in order to assess whether one single score component was driving the association between the score and the MetS, alternative score was calculated by excluding, one at a time, each score component. All reported *P*-values were based on two-sided tests and compared to a significance level of 5%.

## 3 Results

The frequency distribution of the score was symmetrical and looked normal, with only a small percentage of individuals scoring the extreme values of the score (range 15–50) (additional Fig. 1). Table 1 presents descriptive characteristics of the study cohort by quartiles of MedTypeDiet score. Individuals adherent to a Mediterranean-type diet were more likely to be younger, with higher educational status, physical activity level, and non-smokers (Table 1). No significant differences between women and men were observed in relation to the MedTypeDiet score.

The frequency distribution of the score was symmetrical and looked normal, with only a small percentage of individuals scoring the extreme values of the score (range 15–50) (additional Fig. 1). Table 1 presents descriptive characteristics of the study cohort by quartiles of MedTypeDiet score. Individuals adherent to a Mediterranean-type diet were more likely to be younger, with higher educational status, physical activity level, and non-smokers (Table 1). No significant differences between women and men were observed in relation to the MedTypeDiet score.

The association between adherence to a Mediterranean-type diet and various metabolic parameters was tested and shown in Table 2. The analysis of standardized residuals against fitted values did not indicate any discrepancy from homoscedasticity and the assumption of linearity was confirmed by plotting the residuals versus the predicted values (data not shown). Significant associations between the Mediterranean-type diet score and the outcome were observed for WC (β = − 0.296, *P* = 0.015), SBP (β = − 0.436, *P* = 0.046), and TG (β = − 0.021, *P* = 0.012).

Significant association between the MedTypeDiet score and MetS was observed (Table 3). A linear effect was found (*P* for trend = 0.008) and individuals in the highest quartile of the MedTypeDiet score were less likely to have MetS compared with those in the lowest quartile (OR: 0.86, 95% CI: 0.75, 0.99). Increase of one standard deviation of the score was associated with 7% less odds of having MetS (OR 0.93, 95% CI: 0.88, 0.97). The individual components of MetS associated with adherence to the Mediterranean diet were mostly central obesity, blood pressure, and TG component. The analysis stratified by gender revealed a stronger association of the diet in men with TG, whereas in women with central obesity (Table 3).

The association between single components of the score and MetS was evaluated, and adjusted analysis revealed that moderate wine consumption, low dairy products intake, and high total unsaturated:saturated FA ratio were significantly and inversely associated with MetS (Table 4). The analysis of alternative scores created by excluding, one at a time, each individual component of the score showed stable results, with significant trends over quartiles for most of the alternative scores, despite none of the categories were significantly associated with the study outcome (Table 5).

## 4 Discussion

In this study we evaluated the association between adherence to a Mediterranean-type diet and metabolic profile in a sample of Polish adults. We found that about 30% of the individuals suffered by metabolic syndrome, confirming previous prevalence rates [27]. We found that increased adherence scores were associated with lower odds of having MetS, and a specific relation with WC, blood pressure and TG. Despite the associations of MedTypeDiet score by quartiles with MetS were not significant for each category, the analysis by 1-SD increase revealed decreased odds of MetS in both men and women. Since a linear trend is demonstrated, the lack of association by quartiles may depend on the low number of individuals with the condition, as indicated by the large confidence intervals.

The present study is unique in assessing this relationship in a European non-Mediterranean population. To the best of our knowledge, only two studies explored the association between a Mediterranean-type diet and components of MetS in individuals living in the US [19,20]. A cross-sectional analysis was conducted on 780 career male firefighters finding a negative association of adherence to a Mediterranean-type diet and MetS, as well as higher HDL-c and lower LDL-c among higher adherent individuals [19]. A prospective study including individuals of the Framingham cohort reported that higher adherence to a Mediterranean-type dietary pattern was significantly association with lower WC, FPG, TG, and higher HDL-c, as well as overall lower incidence of MetS (38.5% compared with 30.1%) [20]. Under experimental conditions, a study conducted in Canada reported that adherence to the Mediterranean diet led to a decrease in TC, LDL-c, and the total/HDL-c ratio compared to a control diet [18]. Among studies exploring specific components of the MetS, one experimental study [28] reported a better lipoprotein profile in US women with metabolic syndrome, and two studies [16,17] emphasized the role of adherence to a Mediterranean-type diet on BMI status and abdominal adiposity, reporting an inverse association with WC in both men and women, especially in Northern European countries [16], and that individuals more adherent to this dietary pattern were 10% less likely to develop overweight or obesity than individuals with low adherence [17]. There is experimental evidence that the effects of the Mediterranean diet on body fat are associated with alteration of plasma adipokine concentration (in particular reducing leptin and increasing plasma adiponectin concentrations), thus influencing sensation of hunger, adjusting energy expenditures, and regulating glucose regulation and fatty acid oxidation [29]. Our study demonstrated an association of a Mediterranean-type diet with WC, but not with BMI. In contrast, we failed in reporting an association between Mediterranean diet adherence and FPG or having diabetes. Despite a recent pooled analysis of prospective studies reported that high adherence to this dietary pattern was associated with a reduced risk of developing type 2 diabetes [30], it is noteworthy that all the aforementioned studies conducted in non-Mediterranean countries, as well as others specifically focused on diabetes still in multiethnic population [31] did not report benefits of high adherence to a Mediterranean-type diet on glucose and insulin metabolism. Reasons for such discordance are not easy to be identified, as they may depend on different Mediterranean diet individual components and quality, or other factors related with lifestyle or dietary habits that may affect diabetes risk and may differ between Mediterranean and non-Mediterranean countries. However, additional research is needed to further investigate such contrasting results.

The novel scoring system demonstrated similar features than the previous developed by Panagiotakos et al. applied in Mediterranean countries [14,24,32]. We slightly modified the scoring system by taking into account several concerns regarding non-Mediterranean countries. Compared with other scores used for non-Mediterranean countries, our scoring system allowed a wider range of points reachable by one person (range 0–60), minimizing the possibility that dietary habits of non-Mediterranean population could mimic a high adherence to the Mediterranean diet as may happen using a 9-point scale for the index. As a result of previous studies [16–18], an increase of 2 points in the score significantly affects several health outcomes of one person, but this approach applied in individuals living in non-Mediterranean countries could be misleading. For instance, potato intake alone could provide a significant ratio of overall vegetable intake if not considered as an individual component (not included among vegetables) and alcohol intake from liqueurs and spirits could easily provide the amount of daily alcohol generally derived by wine in Mediterranean countries. Several components of the Mediterranean diet demonstrate a biological rationale for their efficacy in protecting by MetS. For instance, moderate consumption of red wine has been reported to decrease cardiovascular risk and that the direct effect of red wine's constituents on endothelial cells is mostly favorable in healthy individuals [33]. Fruit and vegetable consumption has been widely reported to exert a protective action against CVD due to their content in antioxidant compounds (i.e., vitamins and polyphenols) [34,35]. Fish intake has been reported to be inversely associated with cardiovascular protection due to its high content of omega-3 PUFA [36]. In general, the resulting high ratio of total unsaturated:saturated FA beneficially affects the plasma lipid profile, improves insulin sensitivity, and reduces blood pressure [37]. Whole grain products and legumes are rich in fiber and improve insulin sensitivity and protect against the development of diabetes by slowing down carbohydrate digestion and decreasing the glycemic index [38]. In contrast, high intakes of red meat have been associated with increased inflammation, risk of diabetes and hypertension [39,40]. In our study, when we analyzed the level of association with MetS of each individual component using the proposed cut-off points of consumption, we only found association with moderate wine intake, low full-fat dairy products consumption, and high total unsaturated:saturated FA ratio. However, after calculating alternative scores by excluding one at a time each component, we demonstrated that the role of individual components was not enough to explain the significant association of the complete score. Consistently with previous evidence regarding outcomes such as MetS [41], a beneficial association of the overall Mediterranean dietary pattern contrasts with the lack of evidence of an important association of each of its individual components. This may depend on the fact that individual components may be associated only when integrated into the overall score, since a dietary index captured the extremes of the nutritional exposures of interest rather than of individual foods or food groups. Our findings strengthen the hypothesis that the overall dietary pattern, rather than individual foods, should be considered as more representative of the effects in the “real-world” of one's dietary habits.

Our study had some limitations. First, because of its cross-sectional nature, the associations retrieved in the study do not indicate causation or causality. The major drawback of this design is the possibility of reverse causation bias, according to which a general healthier lifestyle affects the health status aside the dietary habits. Despite background characteristics were taken into account when adjusting for potential confounders in multivariate analyses, some residual bias may still exist. Another limitation of this study is pertinent to the sample characteristics. As participants have been recruited in an urban area and response rate accounted for about 60%, study population cannot be considered nationally representative, rather characterized by particularly healthy individuals. However, the aim of the study was to evaluate the relation between adherence to a Mediterranean-like diet and metabolic parameters in a non-Mediterranean country, not to estimate its representativeness in the Polish population. The last issue is related to the methodology of dietary assessment, as FFQs may suffer from recall bias, misclassification of food portions, under- or overestimation of foods and nutrients (i.e., due to seasonality). However, reliability of data collected has been already discussed in details in a previous study [42].

## 5 Conclusions

In conclusion, adherence to a dietary pattern remarking the main components of the original Mediterranean diet may decrease the risk of components associated with MetS even among non-Mediterranean populations. Among the various components of the Mediterranean diet, moderate wine intake, low dairy product consumption, and a high total unsaturated:saturated FA ratio may be of particular importance to health.

The following are the supplementary data related to this article.

Table A1 Components and cut-off points of the Mediterranean-type diet (MedTypeDiet) score.

**Fig. A1:**
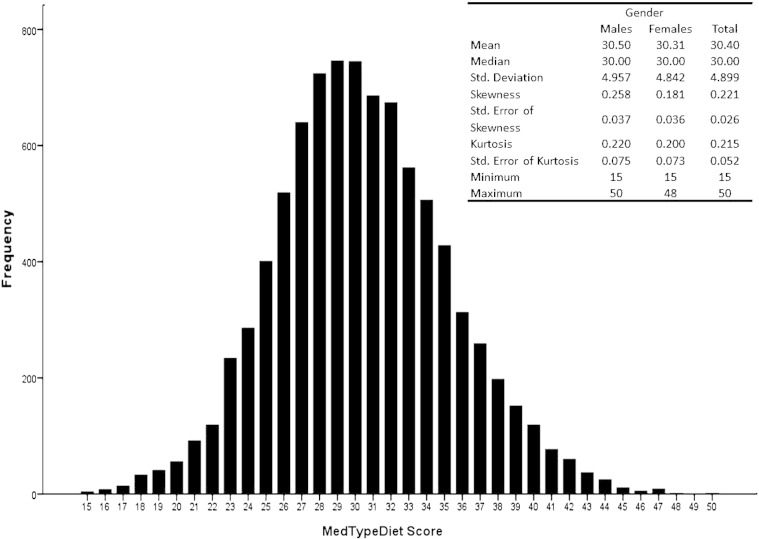
Frequency distribution of the Mediterraean-type diet score among the study sample.

Supplementary data to this article can be found online at http://dx.doi.org/10.1016/j.metabol.2015.02.007.

## Authors' contribution

GG, US, and AP planned study design; GG and AP prepared, drafted, and revised the manuscript; GG, AM, KS performed and revised statistical analysis; US and RTM contributed in data collection; DS and MB provided critical revision of the manuscript.

## Financial disclosure

The study has been funded by the Wellcome Trust (grants 064947/Z/01/Z and 081081/Z/06/Z), US National Institute on Ageing (grant 1R01 AG23522-01) and the MacArthur Foundation Initiative on Social Upheaval and Health (award 71208). Giuseppe Grosso was supported by the International Ph.D. Program in Neuropharmacology, University Medical School of Catania, Catania, Italy.

## Conflict of interest

The authors have no relevant conflict of interest to disclose.

## Figures and Tables

**Table 1:** Background characteristics of the study population by quartiles (Q1–Q4) of MedTypeDiet score and adjusted logistic regression analysis of factors associated with high adherence to the Mediterranean diet.

	MedTypeDiet score				Adjusted^a^ OR (95% CI) of high adherence^b^
	Q1	Q2	Q3	Q4	
MedTypeDiet score, mean (SD)	24.7 (2.3)	29 (0.8)	31.9 (0.8)	36.8 (2.7)	-
Gender, n (%)					
Male	1173 (47.7)	1086 (49.0)	935 (48.6)	1097 (49.5)	1
Female	1287 (52.3)	1132 (51.0)	990 (51.4)	1121 (50.5)	0.98 (0.90, 1.08)
Age group, n (%)					
< 50	438 (17.8)	388 (17.5)	339(17.6)	459 (20.7)	1
51–54	494 (20.1)	471 (21.2)	403 (20.9)	439 (19.8)	0.85 (0.74, 0.98)
55–59	500 (20.3)	485 (21.9)	400 (20.8)	464 (20.9)	0.88 (0.76, 1.01)
60–64	504 (20.5)	447 (20.2)	379 (19.7)	432 (19.5)	0.85 (0.73, 0.98)
≥ 65	524 (21.3)	427 (19.3)	404 (21.0)	424 (19.1)	0.88 (0.75, 1.01)
Educational level, n (%)					
Low	301 (12.2)	288 (13.0)	197 (10.2)	202 (9.1)	1
Medium	1508 (61.3)	1309 (59.0)	1200(62.4)	1237 (55.8)	1.24 (1.07, 1.44)
High	650 (26.4)	620 (28.0)	525(27.3)	778(35.1)	1.44 (1.20, 1.74)
Occupational level, n (%)					
Low	1304 (54.4)	1217 (56.4)	1003 (53.4)	1070 (49.7)	1
Medium	842 (35.1)	709 (32.9)	666 (35.4)	808 (37.5)	1.02 (0.91, 1.14)
High	253 (10.5)	231 (10.7)	211 (11.2)	274 (12.7)	1.02 (0.85, 1.20)
Smoking status, n (%)					
Current smoker	774 (31.5)	692 (31.3)	566 (29.5)	614 (27.7)	1
Non smoker	1680 (68.5)	1520 (68.7)	1354 (70.5)	1599 (72.3)	1.13 (1.02, 1.25)
Alcohol drinking, n (%)					
< 12 g/d	2367 (96.2)	2125 (95.8)	1847 (95.9)	2103 (94.8)	1
> 12 g/d	93 (3.8)	93 (4.2)	78 (4.1)	115 (5.2)	1.15 (1.08, 1.43)
Physical activity, n (%)					
Low	741 (31.8)	628 (29.9)	515 (28.5)	516 (24.2)	1
Moderate	801 (34.4)	735 (35.0)	648 (35.9)	862 (40.5)	1.25 (1.12, 1.39)
High	789 (33.8)	739 (35.2)	643 (35.6)	750 (35.2)	1.20 (1.07, 1.34)

SD, standard deviation.

a Adjusted for age, gender, education, occupation, physical activity, smoking status, alcohol drinking, and total energy intake.

b Highest vs. other quartiles.

**Table 2:** Metabolic parameters of the study population by category of MedTypeDiet score and multivariate linear regression analyses (beta and standard error)^a^ with the score considered as continuous variable.

	MedTypeDiet score				Changes associated with 1-SD increment, β (SE)^a^	*P*
	Q1	Q2	Q3	Q4		
	Mean (SD)					
BMI	28.12 (4.63)	28.18 (4.70)	28.11 (4.58)	28.00 (4.45)	− 0.018 (0.049)	0.717
WC (cm)	92.91 (12.48)	92.56 (12.39)	92.32 (12.32)	92.10 (12.28)	− 0.296 (0.122)	0.015
SBP (mmHg)	138.69 (20.88)	138.23 (21.13)	138.76 (21.83)	136.89 (20.77)	− 0.436 (0.218)	0.046
DBP (mmHg)	86.26 (11.90)	86.35 (11.70)	86.30 (12.02)	85.82 (11.56)	− 0.186 (0.129)	0.147
FPG (mmol/L)	5.37 (1.41)	5.36 (1.97)	5.38 (1.48)	5.34 (1.42)	0.010 (0.015)	0.522
TC (mmol/L)	5.82 (1.07)	5.83 (1.05)	5.79 (1.06)	5.81 (1.06)	− 0.007 (0.012)	0.540
HDL-c (mmol/L)	1.43 (0.38)	1.44 (0.38)	1.44 (0.38)	1.44 (0.37)	0.004 (0.004)	0.279
LDL-c (mmol/L)	3.64 (0.95)	3.64 (0.94)	3.63 (0.95)	3.65 (0.95)	− 0.002 (0.011)	0.849
TGC (mmol/L)	1.60 (0.78)	1.61 (0.78)	1.57 (0.72)	1.55 (0.73)	− 0.021 (0.008)	0.012
TC:HDL ratio	4.29 (1.33)	4.26 (1.21)	4.27 (1.38)	4.25 (1.42)	− 0.019 (0.015)	0.185

BMI, body mass index; DBP, diastolic blood pressure; FPG, fasting plasma glucose; LDL-c, low-density lipoprotein cholesterol; HDL-c, high-density lipoprotein cholesterol; SBP, systolic blood pressure; SD, standard deviation; SE, standard error; TC, total cholesterol; TGC, triglycerides; WC, waist circumference.

a Adjusted for age, gender, education, occupation, physical activity, smoking status, alcohol drinking, and total energy intake.

**Table 3:** Multivariate adjusted odds ratios (95% confidence interval)^a^ for metabolic syndrome and its individual components by category of MedTypeDiet score and 1-standard deviation (SD) increment, overall and by gender.

	MedTypeDiet score, OR (95% CI)^a^				*P* for trend	1-SD score increment, OR (95% CI)^a^
	Q1	Q2	Q3	Q4		
Metabolic syndrome						
Overall	1	0.95 (0.83, 1.09)	0.94 (0.81, 1.08)	0.86 (0.75, 0.99)	0.008	0.93 (0.88, 0.97)
Men	1	0.98 (0.81, 1.19)	0.90 (0.73, 1.10)	0.91 (0.74, 1.11)	0.042	0.93 (0.86, 0.99)
Women	1	0.97 (0.80, 1.19)	0.91 (0.75, 1.10)	0.82 (0.68, 0.99)	0.048	0.91 (0.85, 0.98)
WC (≥ 90 cm in men, ≥ 80 cm in women)						
Overall	1	0.89 (0.78, 1.01)	0.86 (0.75, 0.98)	0.85 (0.75, 0.97)	0.003	0.95 (0.91, 0.99)
Men	1	0.99 (0.82, 1.20)	0.85 (0.69, 1.03)	0.96 (0.80, 1.16)	0.681	0.97 (0.91, 1.03)
Women	1	0.80 (0.67, 0.95)	0.87 (0.73, 1.05)	0.76 (0.64, 0.91)	0.067	0.91 (0.85, 0.97)
SBP (≥ 130 mmHg) or DBP (≥ 85 mmHg or hypertensive treatment)						
Overall	1	0.95 (0.84, 1.08)	1.01 (0.88, 1.16)	0.89 (0.78, 1.01)	0.062	0.95 (0.91, 0.99)
Men	1	1.05 (0.87, 1.26)	1.02 (0.84, 1.24)	0.89 (0.74, 1.08)	0.338	0.93 (0.87, 1.00)
Women	1	0.86 (0.72, 1.03)	0.99 (0.82, 1.19)	0.88 (0.74, 1.05)	0.324	0.94 (0.88, 1.01)
HDL-c (< 40 mg/dl in men, < 50 mg/dl in women)						
Overall	1	0.98 (0.85, 1.14)	1.01 (0.87, 1.17)	0.91 (0.79, 1.05)	0.238	0.97 (0.92, 1.02)
Men	1	1.01 (0.81, 1.25)	1.04 (0.83, 1.30)	0.98 (0.79, 1.22)	0.844	0.98 (0.91, 1.06)
Women	1	0.95 (0.78, 1.15)	0.96 (0.78, 1.15)	0.84 (0.69, 1.03)	0.194	0.95 (0.88, 1.02)
TG (≥ 150 mg/dl)						
Overall	1	1.04 (0.91, 1.17)	0.90 (0.79, 1.03)	0.90 (0.79, 1.02)	0.019	0.94 (0.90, 0.98)
Men	1	1.04 (0.87, 1.24)	0.91 (0.76, 1.10)	0.84 (0.70, 0.99)	0.012	0.90 (0.85, 0.96)
Women	1	1.03 (0.86, 1.24)	0.90 (0.74, 1.09)	0.99 (0.83, 1.19)	0.348	0.97 (0.91, 1.04)
FPG (≥ 100 mg/dl or diabetes treatment)						
Overall	1	0.97 (0.80, 1.19)	0.94 (0.76, 1.16)	0.98 (0.80, 1.21)	0.442	0.97 (0.91, 1.05)
Men	1	1.08 (083, 1.41)	1.00 (0.75, 1.32)	1.01 (0.77, 1.33)	0.835	0.99 (0.91, 1.09)
Women	1	0.84 (0.62, 1.15)	0.88 (0.64, 1.22)	0.97 (0.71, 1.32)	0.304	0.93 (0.83, 1.05)

DBP, diastolic blood pressure; FPG, fasting plasma glucose; HDL-c, high-density lipoprotein cholesterol; SBP, systolic blood pressure; TG, triglycerides; WC, waist circumference.

a Adjusted for age, gender (except when analyses were stratified by sex), education, occupation, physical activity, smoking status, alcohol drinking, and total energy intake.

**Table 4:** Multivariate adjusted odds ratios (95% confidence interval) a for metabolic syndrome by proposed cut-off points in the MedTypeDiet score indicating high adherence to the diet (4 or more score points).

	Cut-off points for high adherence	OR (95% CI)^a^
Whole-grain cereals	150 g/d or more	0.94 (0.77, 1.14)
Potatoes	100 g/d or more	1.02 (0.92, 1.15)
Fruits	500 g/d or more	0.96 (0.85, 1.09)
Vegetables	550 g/d or more	1.11 (0.87, 1.40)
Legumes	50 g/d or more	0.97 (0.87, 1.08)
Fish	50 g/d or more	1.01 (0.82, 1.25)
Red meat	90 g/d or less	0.99 (0.88, 1.10)
Poultry	100 g/d or less	0.91 (0.71, 1.17)
Full-fat dairy products	200 g/d or less	0.85 (0.77, 0.94)
Wine	Up to 400 ml	0.80 (0.07, 0.91)
Nuts and seeds	30 g/d or more	0.83 (0.51, 1.37)
Total unsaturated:saturated	1.25 or more	0.81 (0.73, 0.90)
FA ratio		

FA, fatty acids.

a Adjusted for all the food items listed in the table, age, gender (except when analyses were stratified by sex), education, occupation, physical activity, smoking status, alcohol drinking, and total energy intake.

**Table 5:** Multivariate adjusted odds ratios (95% confidence interval) a for metabolic syndrome recalculated by excluding, one at the time, the proposed components of the MedTypeDiet score.

	MedTypeDiet score, OR (95% CI)^a^				*P* for trend	1-SD score increment, OR (95% CI)^a^
MedTypeDiet score recalculated excluding:	Q1	Q2	Q3	Q4		
Whole-grain cereals	1	0.96 (0.84, 1.09)	0.92 (0.80, 1.06)	0.93 (0.80, 1.07)	0.026	0.97 (0.92, 1.02)
Potatoes	1	0.98 (0.87, 1.11)	0.99 (0.85, 1.14)	0.89 (0.76, 1.04)	0.027	0.97 (0.92, 1.02)
Fruits	1	0.94 (0.83, 1.07)	0.92 (0.81, 1.06)	0.89 (0.77, 1.03)	0.011	0.97 (0.92, 1.02)
Vegetables	1	0.95 (0.83, 1.08)	0.97 (0.84, 1.11)	0.91 (0.79, 1.05)	0.037	0.97 (0.92, 1.02)
Legumes	1	0.89 (0.79, 1.02)	1.01 (0.87, 1.16)	0.89 (0.76, 1.05)	0.050	0.97 (0.92, 1.02)
Fish	1	0.94 (0.83, 1.07)	0.93 (0.81, 1.06)	0.89 (0.77, 1.03)	0.011	0.97 (0.92, 1.02)
Red meat	1	0.98 (0.87, 1.11)	0.98 (0.85, 1.14)	0.93 (0.79, 1.08)	0.028	0.98 (0.93, 1.03)
Poultry	1	0.99 (0.88, 1.13)	1.03 (0.89, 1.20)	0.90 (0.76, 1.07)	0.117	0.97 (0.93, 1.02)
Full-fat dairy products	1	0.97 (0.87, 1.09)	0.94 (0.81, 1.09)	0.87 (0.74, 1.03)	0.011	0.99 (0.93, 1.03)
Wine	1	0.99 (0.86, 1.14)	0.91 (0.79, 1.05)	0.97 (0.83, 1.12)	0.277	0.99 (0.95, 1.05)
Nuts and seeds	1	0.98 (0.85, 1.13)	1.00 (0.87, 1.16)	0.93 (0.81, 1.07)	0.118	0.98 (0.93, 1.03)
Total unsaturated:saturated	1	1.03 (0.90, 1.16)	0.91 (0.79, 1.05)	0.89 (0.76, 1.03)	0.003	0.95 (0.91, 1.01)
FA ratio						

FA, fatty acids.

a Adjusted for age, gender (except when analyses were stratified by sex), education, occupation, physical activity, smoking status, alcohol drinking, and total energy intake.
