# Supplementary material for: A Mediterranean-type diet is associated with better metabolic profile in urban Polish adults: Results from the HAPIEE study
Source: Metabolism. 2015 Jun;64(6):738–46. doi: 10.1016/j.metabol.2015.02.007 (PMC4411218; doi:10.1016/j.metabol.2015.02.007)
Supplement: Table A1 — Components and cut-off points of the Mediterranean-type diet (MedTypeDiet) score. [file mmc1.docx]

Table A1. Components and cut-off points of the Mediterranean-type diet (MedTypeDiet) score.

|  | Score | | | | | |
| --- | --- | --- | --- | --- | --- | --- |
|  | 5 | 4 | 3 | 2 | 1 | 0 |
| Cereals | >200 gr/d | 150-200 gr/d | 60-149 gr/d | 25-59 gr/d | 1-24 gr/d | 0 or rarely |
| Potatoes | >150 gr/d | 100-150 gr/d | 40-99 gr/d | 15-39 gr/d | 1-14 gr/d | 0 or rarely |
| Fruits | >600 gr/d | 500-600 gr/d | 300-499 gr/d | 100-299 gr/d | 1-100 gr/d | 0 or rarely |
| Vegetables | >650 gr/d | 550-650 gr/d | 350-549 gr/d | 150-349 gr/d | 1-100 gr/d | 0 or rarely |
| Legumes | >80 gr/d | 50-80 gr/d | 30-49 gr/d | 10-29 gr/d | 1-9 gr/d | 0 or rarely |
| Fish | >80 gr/d | 50-80 gr/d | 30-49 gr/d | 10-29 gr/d | 1-9 gr/d | 0 or rarely |
| Meat products | <80 gr/d | 80-90 gr/d | 91-120 gr/d | 121-160 gr/d | 161-200 gr/d | >200 gr/d |
| Poultry | <90 gr/d | 90-100 gr/d | 101-110 gr/d | 111-120 gr/d | 121-200 gr/d | >200 gr/d |
| Dairy products | <150 gr/d | 150-200 gr/d | 201-300 gr/d | 301-400 gr/d | 401-500 gr/d | >500 gr/day |
| Wine | <300 ml | 300-400 ml | 401-500 ml | 501-600 ml | 601-700 ml | >700 or 0 ml |
| Nuts and seeds | >40 gr/d | 30-40 gr/d | 20-29 gr/d | 10-19 gr/d | 1-9 gr/d | 0 or rarely |
| Total unsaturated:saturated FA ratio | Quantile 6 | Quantile 5 | Quantile 4 | Quantile 3 | Quantile 2 | Quantile 1 |
| FA, fatty acids. | | | | | | |
